# Effect of Pre-Emulsified Flaxseed Oil Containing Rutin on the Quality of *Nemipterus virgatus* Surimi Gel: Gelatinization Properties, Storage Stability, and Protein Digestibility

**DOI:** 10.3390/foods14020242

**Published:** 2025-01-14

**Authors:** Qingguan Liu, Xiaobing Huang, Huanta Ma, Xinyi Qin, Pengzhi Hong, Xiaowen Pi, Chunxia Zhou

**Affiliations:** 1 College of Food Science and Technology, Guangdong Ocean University, Guangdong Provincial Key Laboratory of Aquatic Product Processing and Safety, Guangdong Provincial Engineering Technology Research Center of Marine Food, Guangdong Modern Agricultural Science and Technology Innovation Center, Zhanjiang 524088, China; liuqingguansdk@163.com (Q.L.); huang14795223331@163.com (X.H.); 17876042414@163.com (H.M.); hongpengzhi@126.com (P.H.); 2 Southern Marine Science and Engineering Guangdong Laboratory (Zhanjiang), Zhanjiang 524088, China; 3 College of Food Science, Southwest University, Chongqing 400715, China

**Keywords:** surimi, pre-emulsified flaxseed oil, rutin, gelatinization properties, storage stability, digestive characteristics

## Abstract

Rinsing during surimi protein processing can result in the loss of essential nutrients, such as fats and minerals. Therefore, supplementing functional fats in a stable form can make up for the fat loss of surimi during the rinsing process. This research aimed to investigate the effects of incorporating pre-emulsified flaxseed oil with different concentrations of rutin (0, 0.5, 1.5, 2.5, and 3.5%, dissolved in flaxseed oil, *w*/*v*) to *Nemipterus virgatus* surimi on the gelatinization properties, lipid oxidation, and in vitro static simulated digestion characteristics of surimi gels. The results indicated that the addition of 1.5% rutin significantly improved the water-holding capacity and decreased the cooking loss rate of surimi gel (*p* < 0.05). The results of optical microscopy and scanning electron microscopy showed that the addition of 1.5% rutin promoted a denser network structure of surimi gel. Furthermore, the incorporation of rutin effectively slowed lipid oxidation in pre-emulsified flaxseed oil surimi gel. Compared with the gel group containing only pre-emulsified flaxseed oil, the addition of rutin significantly reduced the levels of volatile base nitrogen (TVB-N) and thiobarbituric acid reactive substances (TBARSs) in the gel and also mitigated the decline in acidity (*p* < 0.05). Moreover, the addition of rutin significantly inhibited the decrease in pH of surimi gel during storage (*p* < 0.05). In vitro static simulated digestion demonstrated that the addition of 1.5% rutin enhanced the protein digestibility from 71.2% to 77.2% of the surimi gel. Therefore, adding pre-emulsified oil containing an appropriate amount of rutin to surimi can not only compensate for the fat loss during the surimi rinsing process but also effectively improve the quality characteristics of surimi gels. This research will provide a theoretical basis for the effective addition of functional lipids in surimi products and the development of nutritious and healthy surimi products.

## 1. Introduction

Surimi is an aquatic food raw material that transforms into a slimy fish paste after the washing and chopping of fish [1]. Briefly speaking, fresh fish has to undergo steps like pretreatment (cleaning and removing the head, tail, scales, and internal organs), meat extraction, rinsing, dehydration, fine filtration, chopping, and packaging before it can be made into surimi. It offers several advantages, including (Ⅰ) high protein content, (Ⅱ) low calories, and (Ⅲ) a delicate taste. However, the washing process often removes fish oils rich in polyunsaturated fatty acids (PUFAs). To address this issue, oils or fats, including perilla oil, flaxseed, algae, and fish oil, are commonly added to surimi products to enhance their texture, color, flavor, and nutritional value [2].

In the human body, energy and certain fat-soluble vitamins are provided by the intake of essential fatty acids from vegetable oils, solid vegetable fats, and animal fats. However, animal fats are often high in saturated fatty acids, and excessive intake can burden health. For instance, while the addition of animal fats such as lard can make surimi products tender, juicy, and flavorful, excessive consumption of saturated fatty acids and cholesterol poses health risks, including obesity and cardiovascular disease [3]. Consequently, vegetable oils and fish oils rich in omega-3 polyunsaturated fatty acids are more aligned with the population’s demand for a healthy diet [4]. Celandine oil and perilla seed oil, for example, not only improve the color of surimi but also confer health benefits [5,6]. However, the direct addition of liquid oil may negatively impact the texture of surimi. Studies have shown that pre-emulsified oils and fats, such as emulsion gel stabilized by tilapia myofibrillar protein and pre-emulsified perilla seed or soybean oil stabilized by myofibrillar protein, can enhance the texture, oxidation stability, and organoleptic qualities of surimi compared with the direct use of liquid oils [5,7].

Flaxseed oil (FO) is particularly rich in omega-3 polyunsaturated fatty acids. It is mainly composed of α-linolenic acid (accounting for approximately 57% of its total fatty acid content), oleic acid, and linoleic acid [8]. Flaxseed oil has various health-beneficial physiological activities, such as alleviating cardiovascular diseases, nourishing nerves, enhancing immunity, and improving kidney protection against hypercholesterolemia [9]. However, oils and fats high in polyunsaturated fatty acids are highly susceptible to oxidation, leading to undesirable flavors that can significantly reduce consumer acceptance [10]. Therefore, the addition of antioxidants has emerged as an effective strategy for enhancing the oxidative stability of oils and fats.

Natural antioxidants, such as phenolic compounds and flavonoids, are being explored to effectively improve the antioxidant capacity of oils and fats, serving as substitutes for synthetic antioxidants [11]. The incorporation of natural antioxidants can delay or inhibit lipid and protein oxidation in processed meat products, thereby enhancing their shelf life and nutritional value [12,13]. For example, the addition of coconut shell extract containing phenolic substances to pre-emulsified fish oil has been shown to inhibit the oxidation rate of fish oil and extend the shelf life of surimi gel [14]. Li et al. [15] reported that rutin and ferulic acid exhibited significant resistance to hydroxyl-radical-induced lipid and protein oxidation in yak meat. This suggests that the oxidative stability of meat products can be enhanced by incorporating pre-emulsified oils containing natural antioxidants. Rutin, a natural flavonoid glycoside, has been extensively studied for its antioxidant potential. Research indicates that rutin is more resistant to lipid oxidation than other phenolic compounds when used in pre-emulsified vegetable oils rich in omega-3 fatty acids [16]. In addition, rutin also has a variety of physiological activities that are beneficial to human health, such as antioxidant, anti-inflammatory, and antiviral effects, and it is one of the important raw materials for the development of functional foods [17]. Given these advantages, rutin can be added as an antioxidant with multiple health effects to pre-emulsified flaxseed oil to inhibit oil oxidation. Furthermore, adding pre-emulsified flaxseed oil containing rutin to surimi to prepare pre-emulsified flaxseed oil containing rutin-filled surimi gel can achieve the purpose of supplementing stable and healthy fats to the rinsed surimi.

## 2. Materials and Methods

### 2.1. Materials

The surimi (AAA grade) with protein content of 17.92% was purchased from a local aquatic Products Co., Ltd. Tapioca starch was obtained from Yuwang Co., Ltd. (Dezhou, China). Rutin (food-grade) and whey protein isolate (WPI, 90% protein content) were obtained from Solebold Co., Ltd. (Beijing, China). Flaxseed oil (food-grade) was sourced from Shandong Luhua Group Co., Ltd. (Shandong, China). All other chemical reagents used were of analytical grade.

### 2.2. Preparation of Pre-Emulsified Flaxseed Oil Containing Rutin

Pre-emulsified flaxseed oil was prepared according to Xie et al. [18] with some modifications. A total of 1 g of WPI was dissolved into 50 mL of deionized water to obtain a WPI solution (2%, *w*/*v*). Different masses of rutin were dissolved into 160 mL of flaxseed oil with concentrations of 0, 0.5, 1.5, 2.5, and 3.5% (*w*/*v*). A total of 100 mL of flaxseed oil containing rutin was combined with 50 mL of WPI solution and homogenized using a homogenizer (IKA T25, Staufen, Germany) at 11,000 rpm for 5 min to obtain pre-emulsified flaxseed oil with different rutin concentrations.

### 2.3. Preparation of Pre-Emulsified Flaxseed Oil Containing Rutin-Filled Surimi Gel

The preparation of surimi gel followed the method described by Huang et al. [19]. The 500 g of surimi was minced by a Stephan chopper at 900 rpm for 1 min. Salt and water were added to achieve mass fractions of 2.5% and 80%, respectively. Tapioca starch was then incorporated to achieve a mass fraction of 12%, and the mixture was chopped and mixed for an additional minute. Finally, pre-emulsified flaxseed oil containing varying rutin concentrations (0, 0.5, 1.5, 2.5, and 3.5% in flaxseed oil) was added to the surimi mixture with a mass fraction of 4% and chopped for 3 min. The temperature of the surimi mixture was maintained between 4 °C throughout the chopping and mixing process. The surimi mixture was filled into casings (25 mm) and sealed, followed by two-stage heating (incubated at 40 °C for 30 min, then heated to 90 °C for 20 min) using a water—bath kettle (SHA-C, Changzhou, China) and was finally refrigerated at 4 °C for 12 h. The pre-emulsified flaxseed oil without rutin-filled gel group served as the control. Three parallel samples were prepared for each group.

### 2.4. Analysis of Whiteness

Samples were sliced into thin sections (5 × 20 × 20 mm^3^) using a small knife [20]. The L*, a*, and b* values of the sample were measured using a colorimeter (Model NS800, Shenzhen, China). The calculation equation of whiteness was performed as follows:

(1) $$W=100-\sqrt{{\left(100-L^{*}\right)}^{2}+{a*}^{2}+{b*}^{2}}$$

Among them, *W* represents whiteness, while L*, a*, and b* stand for the values of different aspects of the color space at the sampling time.

### 2.5. Analysis of Texture Properties

Samples were equilibrated at 25 °C and cut into cylinders (20 mm × 20 mm) using a small knife and assessed using a texture analyzer (TA-XTplus C, Godalming, UK). Gel strength was measured using a P/0.5 cylinder, and texture profile analysis (TPA) was performed using a P/0.5S spherical plunger for detecting hardness, springiness, cohesiveness, gumminess, and chewiness [19]. The program was set as follows: the speed before the test was 5.0 mm/s, the speed during and after the measurement was 1.0 mm/s, the strain was 50%, and the trigger force was 5.0 g. Six samples were measured for each treatment.

### 2.6. Analysis of Water Holding Capacity (WHC)

Samples were cut to a thickness of 3 mm and weighed (*M*_1_) accurately. The weighed samples were wrapped with filter paper and then centrifuged at 5000× *g* for 10 min at 4 °C. After removing the filter paper, the samples were weighed again (*M*_2_). The water holding capacity of the samples was calculated as follows:

(2) $$WHC/\%=\frac{M_{2}}{M_{1}}\times 100$$

### 2.7. Analysis of Cooking Loss Rate (CLR)

Samples were sliced and weighed (*G*_1_) accurately in a cooking bag and then heated at 90 °C for 20 min. The heated samples were retrieved and stored at 4 °C for 24 h. After wiping off surface moisture, the samples were weighed again (*G*_2_) [19]. The calculation formula for the cooking loss rate was as follows:

(3) $$CLR/\%=\frac{G_{1}-G_{2}}{G_{1}}\times 100$$

### 2.8. Analysis of Light Microscopic

Light microscopic observation of samples was conducted according to our previous study [19]. The morphological structure of the gel was observed using a fluorescence microscope with a magnification of 200×.

### 2.9. Analysis of Scanning Electron Microscopy (SEM)

SEM observation was conducted according to Li et al. [21] with some modifications. The electron micrographs (magnification of 15,000×) of the various samples were captured using an SEM (JEM-7610-F, Tokyo, Japan) with an accelerating voltage of 8 kV.

### 2.10. Determination of Storage Stability

The surimi gel was stored at 4 °C, and the thiobarbituric acid reactive substances (TBARS), volatile base nitrogen (TVB-N) value, and pH value were measured on days 0, 3, 6, 9, 12, 15, 18, 21, and 28.

#### 2.10.1. Determination of the Oxidation of Oils and Fats

Lipid oxidation of the gels was assessed by determining the TBARS content [22]. The 5 g of the minced gel sample was added to 25 mL of a solution containing 7.5% trichloroacetic acid and 0.1% disodium ethylenediaminetetraacetic acid, centrifuged (8000 rpm for 15 min), and 5 mL of the supernatant was reacted with 5 mL of 0.02 mol/L thiobarbituric acid at 90 °C for 30 min. Absorbance was measured with a spectrophotometer (UV757CRT, Shanghai Jingke Instrument Co., Ltd., Shanghai, China) at 532 nm. The TBARS value was expressed as the mass of malondialdehyde per 1 kg of gel (mg/kg). The formula for calculating the TBARS content is as follows:

TBARS = A_532_ × 0.78 (4)

#### 2.10.2. Determination of TVB-N Content in Surimi Gel

The determination method for TVB-N value was based on Jiang et al. [23], with slight modifications. The 10 g of the sample was mixed with 75 mL of water, shaken well, and stood for 30 min. The 1 g of magnesium oxide was added, and the total nitrogen content in the surimi gel was determined using a semi-automatic Kjeldahl nitrogen meter (Kjeltec™ 8200, Fushua (Beijing, China) Technology and Trade Co., Ltd., Beijing, China).

#### 2.10.3. Determination of pH Value in Surimi Gel

The 5 g of the surimi gel was crushed, and 45 mL of distilled water was added. The mixture was extracted for 30 min, and the pH of the filtrate was measured.

### 2.11. Determination of In Vitro Protein Digestibility

In vitro static simulated digestion of the protein was determined following the method of Le Roux et al. [24] with modifications. Given that surimi gel is solid, it undergoes three stages of digestion: mouth, stomach, and intestines. Recommended electrolyte concentrations for simulated salivary fluid (SSF), simulated gastric fluid (SGF), and simulated intestinal fluid (SIF) were based on human in vivo data as detailed by Minekus et al. [25].

Mouth Digestion Stage: a total of 15 g of surimi was weighed and minced, and then 14 mL of simulated oral digestion fluid was added, with magnetic stirring for 2 min. The 1 mL of amylase solution (enzyme activity of 75 U/mL, prepared by SSF) and 75 μL of 0.3 mol/L CaCl_2_ solution were added, stirred magnetically for 2 min, pH adjusted to 7.0, followed by shaking in a water bath at 37 °C for 2 min, and cooled with ice for 2 min.

Gastric Digestion Stage: A total of 29 mL of SGF was mixed with the mouth digestion mixture, along with 1 mL of pepsin solution (enzyme activity of 2000 U/mL, prepared with SGF) and 15 μL of 0.3 mol/L CaCl_2_ solution. The mixture was stirred magnetically for 2 min, pH adjusted to 3.0, incubated in a water bath at 37 °C for 2 h and then cooled with ice for 2 min.

Intestinal Digestion Stage: A total of 29 mL of SIF was added to the mixture from the gastric digestion stage, along with 1 mL of trypsin (enzyme activity of 100 U/mL, prepared by SIF), 120 μL of 0.3 mol/L CaCl_2_ solution, and 2 mL of 3.1 mmol/L bile salt. The mixture was stirred magnetically for 2 min, pH adjusted to 7.0, and incubated in a water bath at 37 °C for 2 h.

The mixed liquid, after simulated digestion through the mouth—stomach—intestine, is placed into a 250 mL centrifuge tube and centrifuged using a centrifuge (7000 rpm, 4 °C) for 20 min, and then the supernatant is taken. Subsequently, the protein content of the supernatant is determined using a semi-automatic Kjeldahl nitrogen analyzer. The calculation formula for the in vitro protein digestibility is as follows:

(5) $$Protein\;digestibility/\%=\frac{v\times c}{M\times n}\times 100$$

Among them, *v* represents the total volume of the supernatant, *c* represents the protein content of the supernatant (centrifuged and determined using a semi-automatic Kjeldahl nitrogen analyzer), *M* represents the total mass of the surimi sample, and *n* represents the protein content of the surimi sample (without centrifugation and determined using a semi-automatic Kjeldahl nitrogen analyzer).

### 2.12. Statistical Analysis

All experiments mentioned above were conducted a minimum of three times, with results presented as the average ± SD derived from these triplicate measurements. The data were subsequently analyzed using one-way ANOVA (Duncan test) through SPSS 27.0 software, with a significance level established at *p* < 0.05. Graphs were plotted using Origin 24 software.

## 3. Results

### 3.1. Whiteness Analysis

In colorimetry, the three commonly used color values are L*, a*, and b*, representing luminance, red/green, and yellow/blue, respectively. An increase in the L* value indicates enhanced brightness. A negative a* value signifies green, while a positive value indicates red. Similarly, a negative b* value denotes blue, and a positive value indicates yellow, with higher b* values indicating greater yellow intensity. Changes in whiteness were observed in surimi gels with varying concentrations of rutin compared with the control (*p* < 0.05). The addition of rutin significantly decreased the L* and a* values of the surimi gel, while the b* value increased progressively (*p* < 0.05). As rutin content increased, both the L* values and the yellow/green values increased (Table 1). The yellow-green color of rutin is closely associated with the whiteness of the surimi gel. These findings align with visual observations of the surimi gel. Similarly, the addition of green tea extract also reduced the whiteness of surimi products [26]. In summary, excessive rutin negatively impacts the whiteness of surimi gel.

### 3.2. Texture Profile Analysis

The textural properties of surimi gels from *Nemipterus virgatus* with different rutin concentrations are presented in Table 2. The addition of rutin at specific levels (0.5% to 1.5%) increased gel strength and chewiness (*p* < 0.05). Gels with 1.5% rutin exhibited a 6.05% increase in chewiness compared to the control (*p* < 0.05). There was minimal effect on springiness and cohesiveness (*p* > 0.05). However, higher rutin concentrations led to significant decreases in gel strength, hardness, gumminess, and chewiness (*p* < 0.05). These results suggest that moderate rutin concentrations can effectively enhance the gel properties of *Nemipterus virgatus* surimi products. This may be attributed to the phenolic hydroxyl and carbonyl groups in rutin, which can form hydrogen bonds with myofibrillar protein molecules, positively affecting gel formation [27]. However, rutin concentrations above 1.5% may hinder gel texture formation due to the self-aggregation of phenolic compounds, reducing protein cross-linking ability [28]. High concentrations of tea polyphenols also interact less efficiently with proteins [29]. Therefore, selecting the appropriate concentration of rutin in pre-emulsified flaxseed oil is crucial for enhancing the quality of surimi gel.

### 3.3. WHC and CLR Analysis

**Figure 1:**
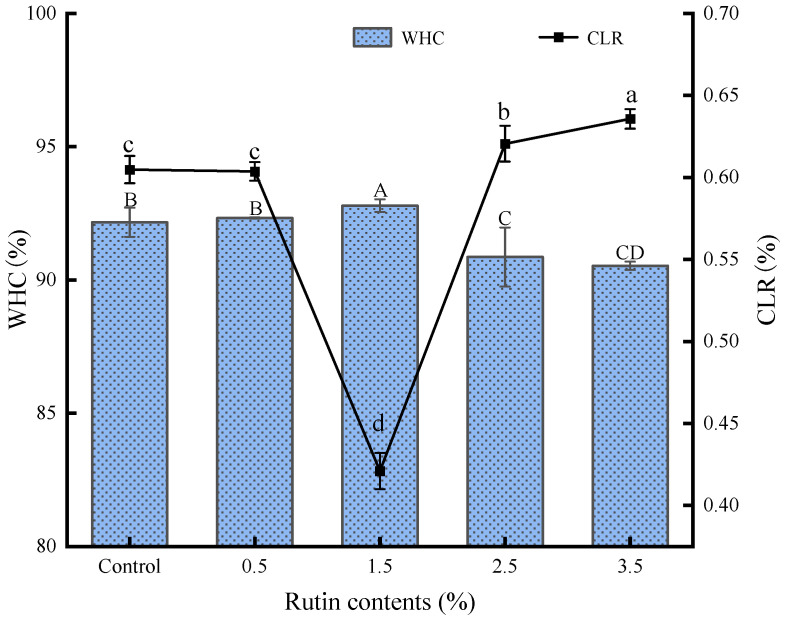
Effects of pre-emulsified linseed oil containing rutin on the water holding capacity (*WHC*) and cooking loss rate (*CLR*) of tapioca starch-*Nemipterus virgatus* surimi composite gels. A–D: different letters indicate differences in *WHC*; a–d: different letters indicate differences in *CLR*.

Water-holding capacity (WHC) and cooking loss rate (CLR) are critical indicators of surimi products’ ability to retain moisture during refrigeration and cooking [2]. The moisture content of surimi gel with different rutin concentrations is illustrated in Figure 1. WHC initially increased and then decreased with increasing rutin addition, while CLR gradually decreased and then increased. Notably, the highest moisture content was observed in gels with 1.50% rutin. Rawel et al. [30] found that moderate levels of phenolic compounds can promote protein molecule cross-linking, resulting in a denser gel structure with higher WHC. Conversely, excessive rutin may lead to entanglement among myofibrillar proteins, causing some proteins to aggregate and lose water-binding capacity [28].

### 3.4. Light Microscopic Observation

**Figure 2:**
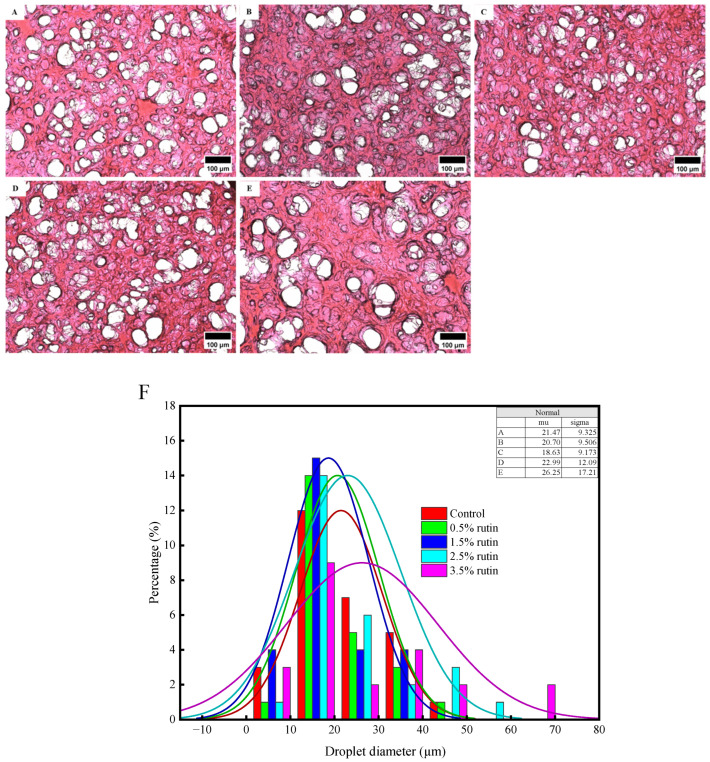
Effects of pre-emulsified flaxseed oil containing rutin on the compactness (**A**–**E**) and cavity diameter (**F**) of surimi gel determined by light microscopic images (200×). A is the control group without rutin, and B–E are samples with 0.5%, 1.5%, 2.5%, and 3.5% of rutin (dissolved in flaxseed oil, *w*/*v*), respectively.

Figure 2 presents a schematic diagram of the optical microscope (A–E) and the corresponding cavity diameter distribution (F), visually illustrating gel structure looseness. Surimi products with 0.5% to 1.5% rutin exhibited a denser gel matrix compared with the control group. Specifically, at a 1.5% addition, the cavity diameter reduced from 22.24 μm to 18.63 μm (Figure 2F). However, rutin concentrations exceeding 1.5% compromised gel network compactness, increasing the cavity diameter to 26.25 μm (Figure 2F). These results indicate that high phenolic concentrations can destabilize surimi protein structures. The appropriate phenolic hydroxyl groups in rutin facilitate protein cross-linking, enhancing gel network compactness. Sharma et al. [31] noted that phenolic compounds can promote protein aggregation through cross-linking. Excessive rutin may hinder disulfide bond formation, leading to a less ordered gel structure.

### 3.5. SEM Observation

**Figure 3:**
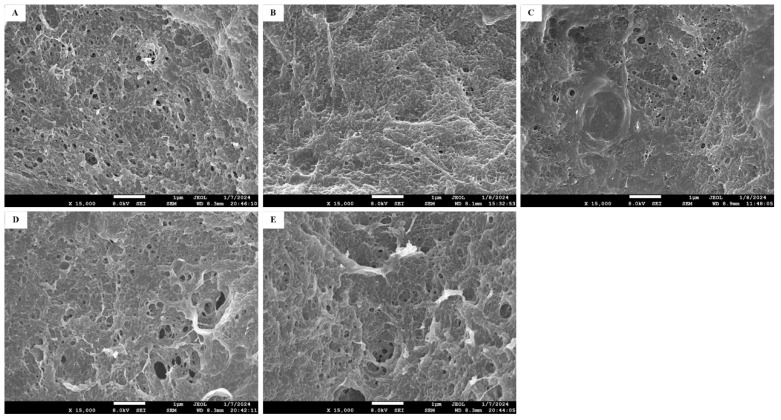
Effects of pre-emulsified flaxseed oil containing rutin on the compactness and cavity diameter of surimi gel determined by scanning electron microscope images (15,000×). (**A**) is the control group without rutin, and (**B**–**E**) are samples with 0.5%, 1.5%, 2.5%, and 3.5% of rutin (dissolved in flaxseed oil, *w*/*v*), respectively.

The quality of surimi products correlates with protein structure, and microstructure reflects gelatinization during heating. Figure 3 illustrates the microstructure of the control gel (without rutin) (A) and gels with 0.50% (B), 1.50% (C), 2.5% (D), and 3.50% (E) rutin. The control group exhibited dense small pores with a rough surface. Gels containing 0.5% and 1.5% rutin exhibited finer, more continuous matrices with improved flatness (Figure 3B,C). This suggests that rutin promotes protein cross-linking, forming dense filaments that polymerize into a fibrous gel network [28]. Phenolic compounds like rutin can cross-link with proteins through hydrophobic interactions and hydrogen bonding, forming a dense network structure [32]. However, concentrations above 1.5% resulted in larger pores in the microstructure (Figure 3D,E), consistent with reduced gel strength, chewiness, and WHC. Balange and Benjakul [28] concluded that high levels of phenolic compounds lead to self-aggregation, impairing protein cross-linking ability and gel network formation.

### 3.6. Storage Stability Analysis

#### 3.6.1. TVB-N Analysis

**Figure 4:**
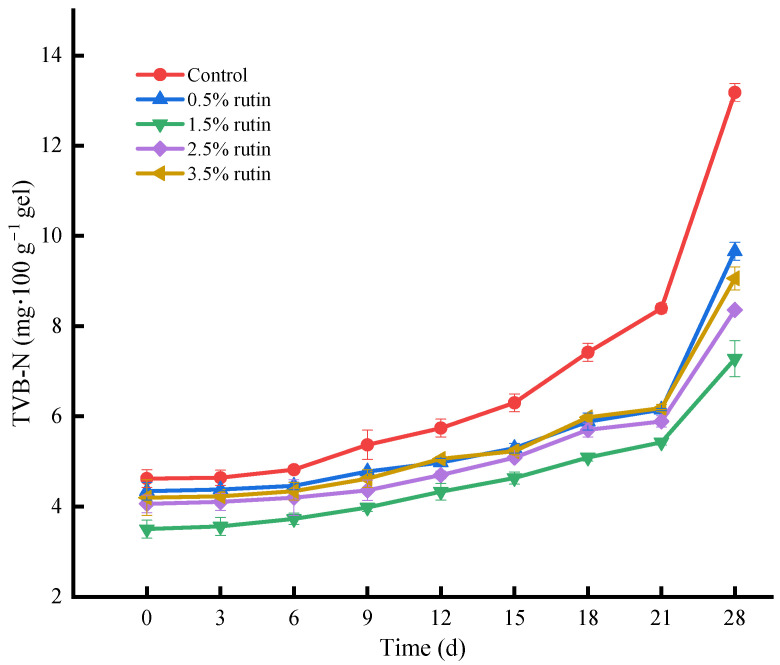
Effects of pre-emulsified flaxseed oil containing rutin on the volatile base nitrogen (TVB-N) content of tapioca starch- *Nemipterus virgatus* surimi composite gels during 28 days storage.

The TVB-N value is a key indicator of aquatic product freshness [33]. Figure 4 illustrates the TVB-N content changes in surimi gel during refrigeration with rutin addition. TVB-N content increased over time across all groups. Rutin significantly inhibited volatile basic nitrogen production, especially in the 1.5% rutin gel group, where TVB-N decreased from 13.18 mg/100 g to 7.28 mg/100 g by the 28th day, indicating the most effective inhibitory effect. Microbial growth leads to protein breakdown into amino acids, producing alkaline nitrogenous substances that elevate TVB-N levels [23]. Rutin effectively inhibits the oxidation of pre-emulsified flaxseed oil and the decomposition of surimi protein. The control group’s TVB-N value rose significantly after 6 days, likely due to rapid enzymatic hydrolysis from oil oxidation, which releases ammonia and other alkaline nitrogen compounds. The antibacterial and antioxidant properties of rutin can inhibit protein decomposition and microbial growth [34]. Notably, the TVB-N content in all groups remained below the national standard of 30 mg/100 g after 28 days of refrigeration.

#### 3.6.2. TBARS Analysis

**Figure 5:**
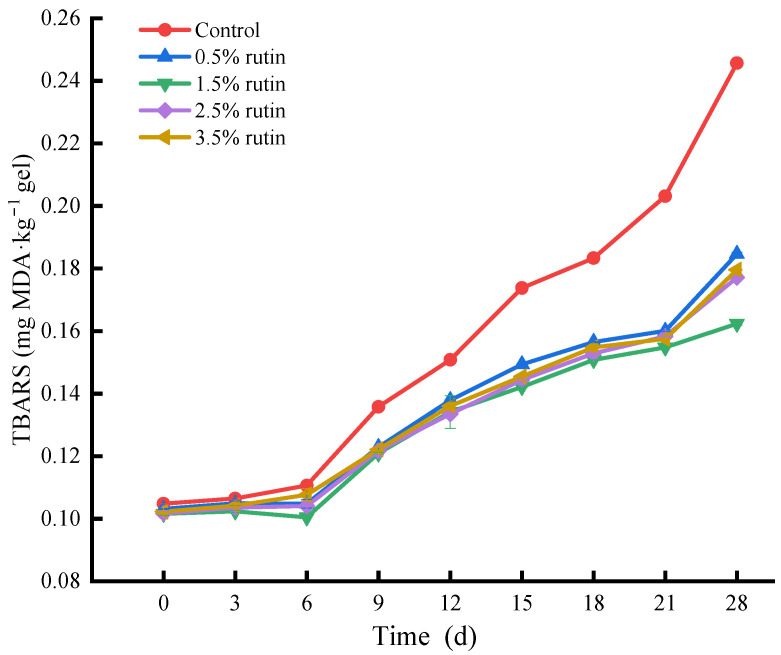
Effects of pre-emulsified flaxseed oil containing rutin on the thiobarbituric acid reactive substances (TBARS) content of tapioca starch-*Nemipterus virgatus* surimi composite gels during 28 days storage.

Malondialdehyde (MDA) is a primary indicator of lipid oxidation. As shown in Figure 5, MDA content in each group remained stable until the sixth day, then increased significantly, likely due to the decomposition of hydroperoxides into secondary oxidation products [14]. Surimi gels with rutin began to exhibit lipid oxidation inhibition at this point. The addition of tea polyphenols has also been effective in inhibiting lipid oxidation [35]. Notably, 1.5% rutin significantly inhibited lipid oxidation after the third day, with MDA content lower than that of the control group without rutin after 28 days. Rutin and ruttyl ester have been reported to inhibit the decomposition of hydroperoxides into secondary oxidation products [36]. This study demonstrated that rutin effectively maintains low MDA levels, with the most significant antioxidant effect observed at a 1.5% concentration.

#### 3.6.3. Surimi Gel pH Analysis

**Figure 6:**
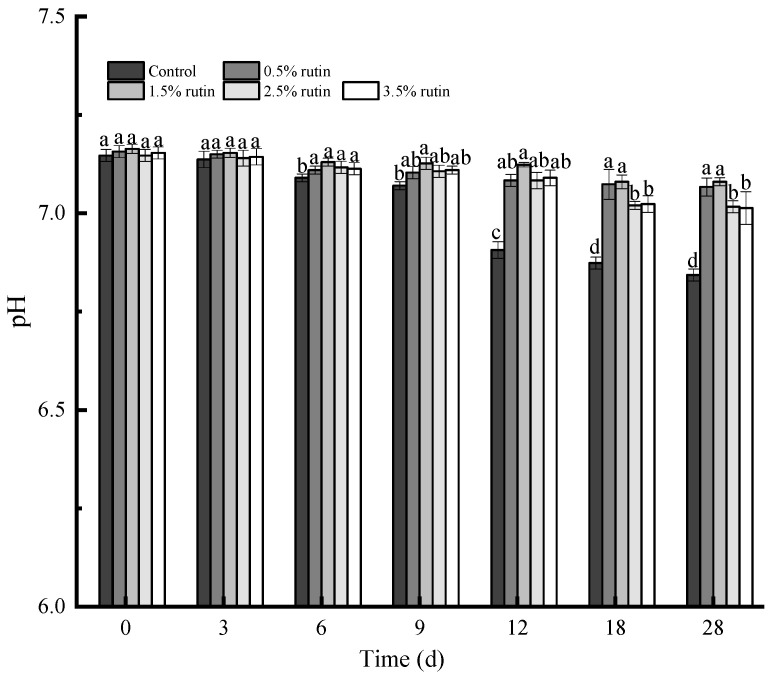
Effects of pre-emulsified flaxseed oil containing rutin on the pH value of tapioca starch–*Nemipterus virgatus* surimi composite gels during 28 days storage. a–d: different letters indicate differences in pH.

Figure 6 illustrates the impact of pre-emulsified flaxseed oil containing rutin on surimi gel pH during refrigeration. Prior to the sixth day, pH values did not change significantly (*p* < 0.05). After the sixth day, only the control group’s pH decreased significantly (*p* < 0.05), while other groups showed a downward trend by the ninth day, except the 1.5% rutin group. After 18 days, the pH values of the two groups without rutin decreased, but not significantly (*p* > 0.05), potentially due to changes in ionic balance in surimi. Similar findings indicated that untreated pork sausages had lower pH during refrigeration [37]. The pH decrease may be attributed to lactate accumulation due to anaerobic glycolysis [38]. By the 18th day, the pH of surimi gel groups with rutin began to decline (*p* < 0.05), but the 0.5% to 1.5% rutin groups exhibited no significant changes throughout storage (*p* > 0.05). Antioxidants like chitosan can slow pH reduction in surimi products [39]. Rutin’s phenolic compounds may inhibit the growth of spoilage microorganisms [40], thereby reducing alkaline amine formation. Overall, rutin enhances the shelf life of surimi products at specific concentrations.

### 3.7. Protein Digestibility Analysis

**Figure 7:**
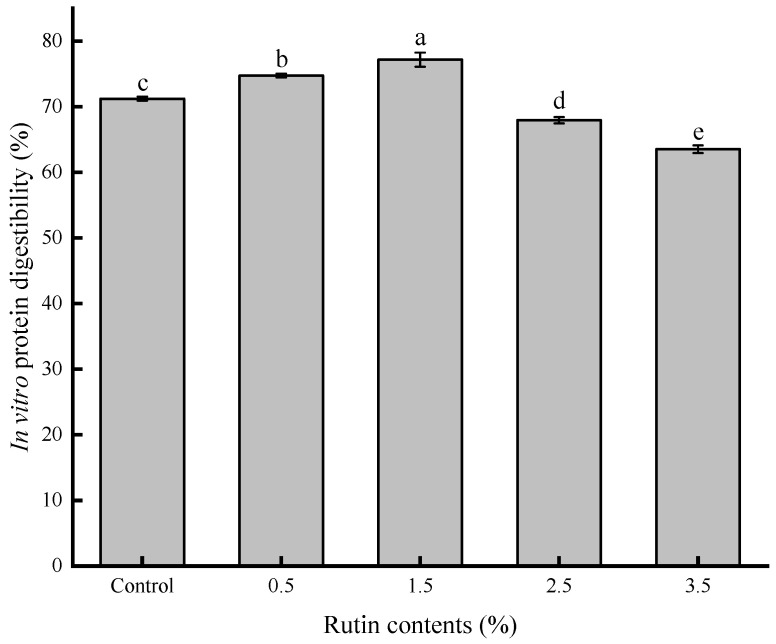
Effects of pre-emulsified flaxseed oil containing rutin on the in vitro digestibility of protein in the tapioca starch-*Nemipterus virgatus* surimi complex gels. a–e: different letters indicate differences in the digestibility of protein.

Surimi gel is rich in high-quality proteins, making its digestibility crucial for gastrointestinal nutrient release [20]. Protein digestibility reflects the extent of surimi protein digestion in the human gastrointestinal tract, with rutin’s effect observed through levels of protein digestion. Compared with the control group, surimi gel protein digestibility initially increased and then decreased with varying rutin concentrations (Figure 7). Notably, at 1.5% rutin, protein digestibility reached 77.18%. Beyond this concentration, digestibility dropped to a minimum of 63.42%. These results suggest that rutin can enhance surimi protein digestion within a certain dosage range. However, exceeding this threshold may hinder absorption and utilization. Appropriate rutin levels can expand the protein molecular structure, promoting covalent cross-linking that facilitates enzymatic digestion by pepsin [41]. Conversely, higher phenolic compound concentrations may resist hydrolysis and protease digestion [42]. Surimi protein in the control group, without rutin, is prone to oxidation, which may impair binding to digestive enzymes and reduce digestibility [43].

## 4. Conclusions

Pre-emulsified flaxseed oil loaded with varying rutin concentrations significantly affected the quality, storage stability, and digestibility of surimi gel. Gel strength, chewiness, and water-holding capacity initially increased and then decreased with rising rutin content, peaking at 1.5%. Low rutin levels can fill voids in the gel reticulum, enhancing gel stability. While rutin negatively affects surimi gel whiteness, it remains within an acceptable range. The rutin-containing gel exhibited lower TBARS and TVB-N values while maintaining a higher pH during refrigeration. Importantly, appropriate rutin concentrations enhance enzymatic digestion by pepsin and improve protein digestibility. Consequently, rutin effectively inhibits lipid oxidation and protein oxidative denaturation in surimi products during storage, positively impacting their shelf life.

## Figures and Tables

**Table 1:** Effects of pre-emulsified flaxseed oil containing rutin on the whiteness of tapioca starch-*Nemipterus virgatus* surimi composite gels.

Samples	L*	a*	b*	Whiteness
Control	73.17 ± 0.27 ^a^	−1.94 ± 0.10 ^a^	5.53 ± 0.24 ^e^	72.54 ± 0.31 ^a^
0.5% rutin	72.88 ± 0.56 ^a^	−2.68 ± 0.02 ^b^	8.09 ± 0.25 ^d^	71.57 ± 0.60 ^b^
1.5% rutin	72.06 ± 0.26 ^b^	−3.43 ± 0.08 ^d^	11.24 ± 0.27 ^c^	69.69 ± 0.14 ^c^
2.5% rutin	71.23 ± 0.13 ^c^	−4.07 ± 0.13 ^e^	14.31 ± 0.21 ^b^	67.61 ± 0.03 ^d^
3.5% rutin	69.92 ± 0.46 ^d^	−4.35 ± 0.07 ^f^	16.11 ± 0.24 ^a^	65.60 ± 0.38 ^f^

*Note*: There is no significant difference if there is a same letter in the same column (*p* > 0.05). Control group without rutin, the concentration of rutin represents the concentration dissolved in flaxseed oil (*w*/*v*).

**Table 2:** Effects of pre-emulsified flaxseed oil containing rutin on the texture properties of tapioca starch-*Nemipterus virgatus* surimi composite gels.

Samples	Strength (N)	Hardness (N)	Springiness	Cohesiveness (g·s)	Gumminess	Chewiness
Control	17.09 ± 0.41 ^b^	24.49 ± 0.78 ^a^	1.41 ± 0.01 ^a^	0.66 ± 0.01 ^a^	16.14 ± 0.63 ^a^	20.67 ± 0.38 ^c^
0.5% rutin	17.05 ± 0.18 ^b^	24.35 ± 0.59 ^a^	1.40 ± 0.01 ^a^	0.66 ± 0.00 ^a^	16.15 ± 0.33 ^a^	21.33 ± 0.44 ^b^
1.5% rutin	17.72 ± 0.12 ^a^	24.31 ± 0.64 ^a^	1.40 ± 0.01 ^a^	0.66 ± 0.00 ^a^	16.01 ± 0.40 ^a^	21.92 ± 0.38 ^a^
2.5% rutin	16.87 ± 0.34 ^b^	23.15 ± 0.10 ^b^	1.30 ± 0.15 ^a^	0.66 ± 0.01 ^a^	15.17 ± 0.21 ^b^	20.94 ± 0.89 ^bc^
3.5% rutin	16.35 ± 0.37 ^c^	23.03 ± 0.27 ^b^	1.29 ± 0.18 ^a^	0.66 ± 0.00 ^a^	15.20 ± 0.19 ^b^	20.58 ± 1.32 ^c^

*Note*: There is no significant difference if there is a same letter in the same column (*p* > 0.05). Control group without rutin, the concentration of rutin represents the concentration dissolved in flaxseed oil (*w*/*v*).

## Data Availability

The original contributions presented in the study are included in the article, further inquiries can be directed to the corresponding author.
